# A cohort study of BMI changes among U.S. Army soldiers during the COVID-19 Pandemic

**DOI:** 10.1186/s12889-023-16460-7

**Published:** 2023-08-15

**Authors:** Marc Wuerdeman, Amanda Banaag, Miranda Lynn Janvrin, Tracey Pérez Koehlmoos

**Affiliations:** 1 Uniformed Services University of the Health Sciences, 4301 Jones Bridge Rd, Bethesda, MD 20814 USA; 2 Henry M. Jackson Foundation for the Advancement of Military Medicine, Inc., 6720 A Rockledge Dr, Bethesda, MD 20817 USA

**Keywords:** Obesity, Military Medicine, Military Health System

## Abstract

**Background:**

The increasing number of individuals with obesity is a healthcare concern in the United States (U.S.) population; the men and women who serve in the Army are no exception, with 17.3% of soldiers categorized with a body mass index (BMI) of Obesity in 2017. The COVID-19 pandemic profoundly disrupted life around the globe. During the pandemic, restrictions to soldier movement and activity were put in place to limit COVID-19 transmission. We strive to assess what effects these changes may have had on the BMIs of soldiers.

**Methods:**

We conducted a retrospective cohort study of active duty U.S. Army soldiers using data from the Military Health System Data Repository. BMI was calculated and categorized before (February 2019 – January 2020) and during the pandemic (September 2020 – June 2021). Women who were pregnant or delivered during and one year prior to the study periods were excluded. Statistical analyses included paired t-tests evaluating mean BMI, percent change, and the Stuart-Maxwell test for marginal homogeneity.

**Results:**

191,894 soldiers were included in the cohort. During the pandemic, 50.5% of soldiers in the cohort were classified as Overweight and 23.2% were classified as Obesity. T-test and Stuart-Maxwell test indicated significant differences and changes in BMI categories between the pre-pandemic and pandemic periods, particularly the Obesity category, which experienced a 5% growth and 27% change. Significant absolute changes were observed during the pandemic; 26.7% of soldiers classified as Healthy weight in the pre-pandemic period shifted to Overweight in the pandemic period and 15.6% shifted from Overweight in the pre-pandemic period to Obesity in the pandemic period. Absolute increases were observed across every demographic category in soldiers with obesity; the categories that saw the highest increases were female, ages 20–24, White, and Junior Enlisted soldiers.

**Conclusions:**

Higher rates of obesity may result in decreased health of the force. The specific needs of younger and Junior Enlisted soldiers need to be further addressed, with focus on special intervention programs by the U.S. Army.

**Supplementary Information:**

The online version contains supplementary material available at 10.1186/s12889-023-16460-7.

## Background

The Coronavirus Disease 2019 (COVID-19) pandemic has been profoundly disruptive to daily life across the United States (U.S.) and the globe. In addition to the potential physiologic effects of the disease itself, the public health protection measures enacted in response to the COVID-19 pandemic have significantly impacted general mental and physical health [1–4]. During the initial response to the pandemic, widespread lockdown, social distancing, and shelter in place measures were enacted [5, 6]. Shelter in place and lockdown orders have been demonstrated to have led to an increase in sedentary activities. Prolonged sedentary activity is associated with increased rates of overweight and obesity [7]. In addition to increases in prolonged sedentary activity, data suggests that these measures have had other significant public health consequences, including increases in weight gain, malnutrition, mental illness, and substance abuse in the general population [1, 2, 8–10].

For the U.S. Army, increases in soldiers with overweight and obesity presents significant public health and readiness concerns [11]. Prior to the COVID-19 pandemic, rates of overweight and obesity among Army soldiers were already rising [12]. In March 2020, the U.S. Army raised the Health Protection Condition (HPCON) level across all installations to Charlie status and placed contingency forces under HPCON Delta to limit further transmission of COVID-19 [13, 14]. As a result, all in-person gatherings were cancelled, travel was either cancelled or restricted, and soldiers were prepared to remain in place for prolonged periods with limited access to services, including indoor fitness and dining facilities [13]. These restrictions may have made it more difficult for soldiers to engage in healthy behaviors. Soldiers with obesity utilize healthcare services at a disproportionately higher rate compared to soldiers with healthy weight or even soldiers with overweight, thus increases in soldiers with obesity poses economic and accessibility challenges for the Military Health System (MHS) [15]. In combination with a health system already under strain from the pandemic, the increase in soldiers with obesity puts added stress on the MHS and threatens deployability and readiness.

This study aims to identify changes in body mass index (BMI) among active-duty U.S. Army soldiers during the COVID-19 pandemic mitigation efforts by the U.S. Department of Defense (DoD) and the U.S. Army. The U.S. Army has strict body fat standards that soldiers must maintain in order to be considered fit for duty and BMI is the most commonly used method by the U.S. Army to estimate body fat [16]. Changes in BMI among soldiers during this time period will indicate if the pandemic lockdown and social distancing measures had any impact on potential military readiness.

## Methods

**Fig. 1:**
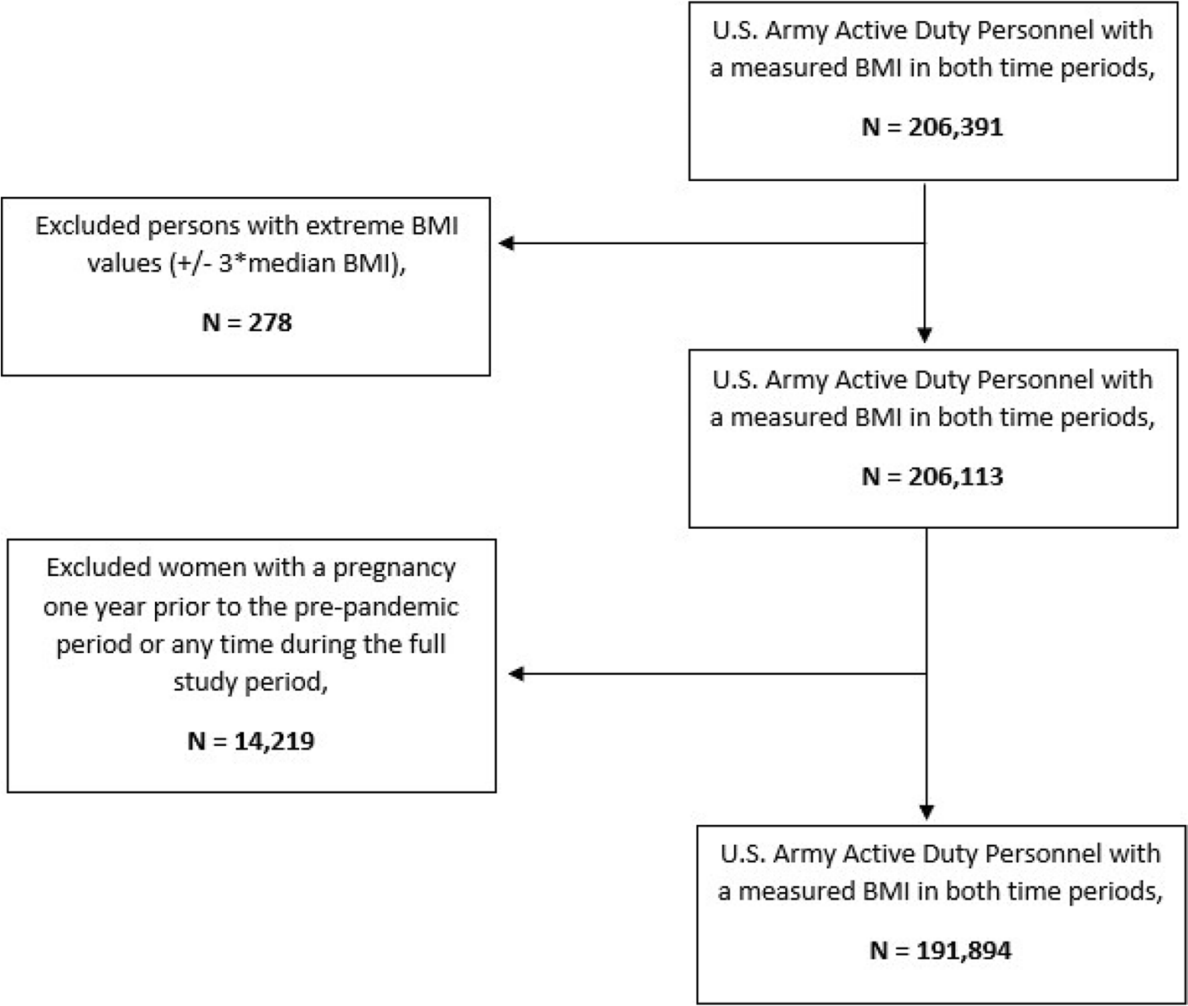
CONSORT Diagram of the U.S. Army Active-Duty Cohort Selection. A CONSORT diagram illustrating application of the inclusion and exclusion criteria to the U.S. Army Active Duty Personnel population to determine the final study population

Using healthcare encounter data from the MHS Data Repository (MDR), we identified a cohort of active-duty U.S. Army soldiers, ages 17 and older and with a reported BMI in both the pre-pandemic period, defined as one year before the DoD’s COVID-19 mitigation efforts were put into place (February 2019 to January 2020), and the pandemic period, defined as September 2020 to June 2021 for this study. The MDR is a central repository of administrative and healthcare data for all MHS beneficiaries who receive care at military treatment facilities (direct care) or at civilian fee-for-service facilities (private sector care) [17]. Personnel in the Army National Guard or reserves (both active and inactive) were excluded from analysis due to inconsistent access to care within the MHS. Additionally, female soldiers who had been pregnant during the year prior or who became pregnant at any time during the study period were also excluded. A CONSORT diagram of those meeting inclusion and exclusion criteria can be found in Fig. 1.

Using vital signs data from the MDR, we calculated BMI from reported height and weight metrics using the Centers for Disease Control & Prevention (CDC) formula for the English System: weight (lbs) / [height (in)]^2^ × 703. BMI categories were classified according to the established CDC guidelines (2022) as follows: Underweight (below 18.5 kg/m^2^), Healthy weight (18.5–24.9 kg/m2), Overweight (25.0–29.9 kg/m2), and Obesity (30.0 kg/m2 and above). To account for any implausible BMI calculations, we used an interquartile method to identify and remove BMI values outside ± 3 standard deviations from the mean. If a soldier had multiple BMI measurements in each observed period (pre-/during pandemic), the most recent measurements were preferentially used to provide measurement snapshots close to the time of DoD COVID-19 mitigation efforts. Due to the availability of necessary height and weight data, BMI was only calculated from in person encounters that were received in the direct care system.

Study analyses including descriptive statistics were performed on patient cohort demographics and stratified by the two observation periods; BMI trends by gender, age groups (5-year increments from < 20 to 50 +), race, and rank category; age and gender adjusted ANCOVA comparing the mean differences in BMIs for the cohort; and percent change in Overweight and Obesity BMI categories from pre-pandemic to during the pandemic. Additionally, we performed the Stuart-Maxwell test of marginal homogeneity to identify statistically significant differences in BMI category distributions over the study period. The Stuart-Maxwell test was chosen based on the paired design of the study, in which each subject's BMI status was assessed at two different time points (pre-pandemic and during the pandemic). Statistical significance was determined as a p-value < 0.05. All statistical analyses were performed using SAS, version 9.4 (SAS Institute Inc., Cary, NC). This research was reviewed and found exempt by the Uniformed Services University Institutional Review Board.

## Results

We identified a cohort of 191,894 active-duty Army soldiers for inclusion in this study. Male soldiers comprised the majority of the cohort at 85.36%, while female soldiers made up the remaining 14.64% (Table 1). The majority of the cohort were Soldiers ages 20–24 years (29.32%) and ages 25–29 years (21.48%), identified as White (66.78%), and in a Junior Enlisted rank (42.91%) (Table 1). This cohort is demographically representative of the U.S. Army active-duty population.

**Table 1:** Cohort Demographics of US Army Active-Duty Service Members, N = 191,894

	**Count**	**Percent of N**
**Gender**		
Female	28,092	14.64
Male	163,802	85.36
**Age Group**		
< 20	16,092	8.39
20–24	56,265	29.32
25–29	41,217	21.48
30–34	28,812	15.01
35–39	25,145	13.10
40–44	13,917	7.25
45–49	7303	3.81
50 +	3143	1.64
**Race**		
White	128,143	66.78
Black	45,336	23.63
Asian/Pacific Islander	14,649	7.63
American Indian/Alaskan Native	1418	0.74
Other	1221	0.64
Missing	1127	0.59
**Rank**		
Junior Enlisted	82,346	42.91
Senior Enlisted	69,270	36.10
Junior Officer	26,039	13.57
Senior Officer	5424	2.83
Warrant Officer	6782	3.53
Other	2033	1.06

Demographic information for the included cohort of Army soldiers

Results from the age and gender adjusted ANCOVA indicate significant increases in the total mean BMI of the cohort and across all BMI categories, excluding those with Obesity [F(11, 55,304) = 238.47, *p* < 0.0001; Table 2]. Rate change analyses indicated the largest change in soldiers with Healthy Weight and soldiers with Obesity, with a 15.8% percent decrease in soldiers classified as Healthy Weight and a 27.3% percent increase in soldiers with Obesity. The results from the Stuart-Maxwell test confirm significant differences in BMI categories between the pre-pandemic and pandemic periods and indicate the direction of change (Table 3). The decrease in soldiers with Healthy weight can be attributed to the 26.7% of soldiers with Healthy weight becoming reclassified as Overweight during the pandemic. The increase in soldiers with Obesity can be attributed to 15.6% of soldiers with Overweight who became reclassified as soldiers with Obesity during this time period.

**Table 2:** Age and Gender Adjusted Comparison of Mean Difference in BMI in Army Soldiers Before and During COVID-19

Pre-Pandemic BMI	N	Mean	95% CL		*p*-value
Underweight	631	3.94	3.65	4.23	< 0.0001
Healthy	59,270	0.53	0.48	0.58	< 0.0001
Overweight	97,036	0.24	0.20	0.29	< 0.0001
Obesity	34,957	-0.49	-0.56	-0.42	< 0.0001

Note: Age and gender adjusted results produced using ANCOVA with Tukey adjustment

**Table 3:** Comparison of Cohort BMI Category Prior to and During COVID-19 Pandemic*

	**BMI Category During COVID-19 Pandemic**			
**BMI Category Pre-COVID19**	**Underweight (*N* = 590)**	**Healthy (*N* = 49,902)**	**Overweight (*N* = 96,904)**	**Obesity (*N* = 44,498)**
**n (percent of row N)**				
**Underweight (*N* = 613)**	155 (24.56)	351 (55.63)	81 (12.84)	44 (6.97)
**Healthy (*N* = 59,270)**	315 (0.53)	42,540 (71.77)	15,849 (26.74)	566 (0.95)
**Overweight (*N* = 97,036)**	87 (0.09)	6749 (6.96)	75,095 (77.39)	15,105 (15.57)
**Obesity (*N* = 34,957)**	33 (0.09)	262 (0.75)	5879 (16.82)	28,783 (82.34)

*p < 0.001 for above table based on Stuart-Maxwell test of marginal homogeneity

Note: We show the pre-pandemic categorization down the y-axis, and follow-up categorization during the pandemic period across the x-axis. From left to right across the table, we show each pre-pandemic BMI group’s absolute changes and percentage shift in the pandemic period

An overview of BMI category for the cohort by the pre-pandemic and during pandemic periods, the absolute changes and percentage shift amongst the BMI categories, and the result from the Stuart-Maxwell test for homogeneity

Supplemental Table 1 shows the unadjusted data looking at the changes of the Obesity and Overweight groups by demographic category. An absolute increase in Obesity was observed across every demographic category. The highest risk demographic groups were Female (52.74%), Ages 20–24 (61.34%), White (30.49%), and Junior Enlisted (60.73%).

## Discussion

We identified a cohort of 191,894 active-duty Army soldiers. Analysis of this cohort indicates that it is demographically representative of the 481,254 soldiers in the Active-Duty Army force according to the Military One Source Demographic 2020 profile [18]. When comparing pre-pandemic and pandemic BMI measurements among the soldiers in our cohort, there were decreases in the number of soldiers with Underweight and Healthy weight and increases in soldiers with Overweight and Obesity. These findings are in line with research by Restrepo et al. that demonstrated increases in average BMI and obesity prevalence among both U.S. adults and children when comparing pre-pandemic and pandemic BMI [10]. Our findings are also align with the studies of active-duty service members by Legg et al. and Stiegmann et al. that demonstrated increases in BMI across the military population in all services [10, 19, 20]. These findings are expected given the increases in prolonged sedentary activity and increase in alcohol consumption that has been shown to have occurred during the pandemic [21, 22].

A 2019 study by Shiozawa et al. indicated that more than 70% of male active duty soldiers were above normal weight in FY2015 [15]. Results from the pandemic period of this study indicate that 73.7% of all active-duty soldiers in our cohort are above what we classify as Healthy weight, up from 68.8% in the pre-pandemic period. Further, 76.5% of male active-duty soldiers are above Healthy weight, up from 71.8% in the pre-pandemic period. Shiozawa et al.’s study emphasized the impact that soldiers with Overweight and Obesity have on the MHS through greater health service utilization and that in every major diagnostic category, soldiers with obesity use disproportionately more services than soldiers with normal weight [15]. Increases in this category in particular may dramatically strain MHS resources.

The largest changes in BMI category were seen in soldiers in the Ages 20–24, and Junior Enlisted categories. These soldiers are often living in the barracks who attend organized physical training and depend on the provided dining facilities to eat [23]. Given that our population is demographically representative of the Army soldier population, it is therefore reasonable to assume that restrictions of movement and activities on installations could have adversely impacted the health of these soldiers in particular.

Strengths of this study include its large demographically representative cohort population and its use of evidence from the medical records of soldiers rather than self-reported data. Additionally, the analyses benefited from longitudinal data with two BMI calculations: one before and one during the pandemic period.

However, this study has limitations. This study is a descriptive study of BMI changes among the U.S. Army population and thus cannot be used to establish a cause and effect relationship between the pandemic and increases in BMI among soldiers. Descriptive studies are nonetheless important additions to the literature as they help to estimate the burden of disease and disease trends in specific populations [24]. It is important to note that this study is based solely on BMI and does not include other metrics such as waist circumference, skinfold measurements, or body fat percentages, which can be misleading for overly muscular individuals, such as those in the military population. Additionally, although body composition may vary by Military Occupational Specialty (MOS), this analysis did not evaluate BMI by MOS. This study is also limited to the U.S. Army population and thus does not include data from the other services and therefore the results are not generalizable to these populations, nor to the general U.S. population. Further, data from the MDR may not be a comprehensive representation of a patient’s medical history that may indicate further medical causes for weight gain, excluding pregnancy, and only captures data for those seeking medical care.

Based on the results from this study and the literature, increases in BMI among Army soldiers are likely to continue unless there is intervention. Future research into targeted measures to prevent obesity among soldiers during future public health emergencies is needed, especially in times of lockdown and social distancing measures. These interventions should address appropriate diet education, nutritious food options, exercise maintenance for soldiers, especially among junior enlisted soldiers. Further research into the impact that post-pandemic obesity has on readiness would help to highlight the importance this issue has in the military community specifically.

## Conclusions

Among the cohort of 191,894 active-duty U.S. Army soldiers included in our study, there were statistically significant increase in mean BMI across all pre-pandemic BMI groups. As a result, the Overweight and Obesity categories comprise 73.7% of the cohort and the prevalence of soldiers with Obesity increased from 18% prior to the COVID-19 pandemic to 23% during, posing a significant public health and military readiness concern.

Further research should assess if similar findings were seen among the other military services and if these effects persisted in the post-pandemic period. Future targeted interventions are needed to prevent obesity among soldiers in the event of future public health emergencies. By developing strategies to combat the pandemic related rise in obesity among soldiers, the Army and DoD could serve as an example for public health recovery and wellness promotion to the nation.

### Supplementary Information

**Additional file 1.**

## Data Availability

The data that support the findings of this study are available from the United States Defense Health Agency. Restrictions apply to the availability of these data, which were used under Federal Data User Agreements for the current study, and so are not publicly available.

## Abbreviations

BMI: Body Mass Index
CDC: Centers for Disease Control and Prevention
COVID-19: Coronavirus Disease 2019
DoD: U.S. Department of Defense
MHS: Military Health System
MDR: Military Health System Data Repository
MOS: Military Occupational Specialty
U.S.: United States
