# Supplementary material for: A cohort study of BMI changes among U.S. Army soldiers during the COVID-19 Pandemic
Source: BMC Public Health. 2023 Aug 15;23:1547. doi: 10.1186/s12889-023-16460-7 (PMC10426164; doi:10.1186/s12889-023-16460-7)
Supplement: Supplementary file 1 — Additional file 1. [file 12889_2023_16460_MOESM1_ESM.docx]

Supplemental Table 1. Demographics of Army Soldiers by Overweight and Obese BMI During COVID-19 Pandemic

| **Cohort Demographics by Overweight and Obese BMI During COVID-19 Pandemic** | | | | | | | | | | |
| --- | --- | --- | --- | --- | --- | --- | --- | --- | --- | --- |
|  | **Pre-Pandemic BMI** | | | | **Pandemic BMI** | | | | **Percent Change of Count** | |
|  | **Overweight (N=97,036)** | | **Obese (N=34,957)** | | **Overweight (N=96,904)** | | **Obese (N=44,498)** | | **Overweight BMI** | **Obese BMI** |
|  | **Count** | **% of Column N** | **Count** | **% of Column N** | **Count** | **% of Column N** | **Count** | **% of Column N** |  |  |
| **Gender** |  | | | |  | | | |  | |
| Female | 11941 | 12.31 | 2448 | 7.00 | 12295 | 12.69 | 3739 | 8.40 | **2.96** | **52.74** |
| Male | 85095 | 87.69 | 32509 | 93.00 | 84609 | 87.31 | 40759 | 91.60 | **-0.57** | **25.38** |
| **Age Group** |  | | | |  | | | |  | |
| <20 | 5592 | 5.76 | 674 | 1.93 | 6658 | 6.87 | 1714 | 3.85 | **19.06** | **154.30** |
| 20-24 | 26320 | 21.12 | 6146 | 17.58 | 26836 | 27.69 | 9916 | 22.28 | **1.96** | **61.34** |
| 25-29 | 21977 | 22.65 | 6804 | 19.46 | 21762 | 22.46 | 8862 | 19.92 | **-0.98** | **30.25** |
| 30-34 | 16099 | 16.59 | 6444 | 18.43 | 15677 | 16.18 | 7547 | 16.96 | **-2.62** | **17.12** |
| 35-39 | 13775 | 14.20 | 7375 | 21.10 | 13220 | 13.64 | 8245 | 18.53 | **-4.03** | **11.80** |
| 40-44 | 7438 | 7.67 | 4465 | 12.77 | 7123 | 7.35 | 4901 | 11.01 | **-4.24** | **9.76** |
| 45-49 | 4023 | 4.15 | 2237 | 6.40 | 3873 | 4.00 | 2430 | 5.46 | **-3.73** | **8.63** |
| 50+ | 1812 | 1.87 | 812 | 2.32 | 1755 | 1.81 | 883 | 1.98 | **-3.15** | **8.74** |
| **Race** |  | | | |  | | | |  | |
| White | 66682 | 68.72 | 20880 | 59.73 | 66788 | 68.92 | 27246 | 61.23 | **0.16** | **30.49** |
| Black | 21260 | 21.91 | 10291 | 29.44 | 20976 | 21.65 | 12854 | 28.89 | **-1.34** | **24.91** |
| Asian/Pacific Islander | 7074 | 7.29 | 3010 | 8.61 | 7134 | 7.36 | 3500 | 7.87 | **0.85** | **16.28** |
| American Indian/Alaskan Native | 746 | 0.77 | 300 | 0.86 | 727 | 0.75 | 385 | 0.87 | **-2.55** | **28.33** |
| Other | 678 | 0.70 | 346 | 0.99 | 676 | 0.70 | 356 | 0.80 | **-0.29** | **2.89** |
| Missing | 596 | 0.61 | 130 | 0.37 | 603 | 0.62 | 157 | 0.35 | **1.17** | **20.77** |
| **Rank Group** |  | | | |  | | | |  | |
| Junior Enlisted | 37259 | 38.40 | 9885 | 28.28 | 38219 | 39.44 | 15888 | 35.70 | **2.58** | **60.73** |
| Senior Enlisted | 37430 | 38.57 | 18043 | 51.61 | 36232 | 37.39 | 20924 | 47.02 | **-3.20** | **15.97** |
| Junior Officer | 14433 | 14.87 | 3945 | 11.29 | 14559 | 15.02 | 4352 | 9.78 | **0.87** | **10.32** |
| Senior Officer | 3ll4 | 3.21 | 1261 | 3.61 | 3038 | 3.14 | 1346 | 3.02 | **-2.44** | **6.74** |
| Warrant Officer | 3973 | 4.09 | 1711 | 4.89 | 3869 | 3.99 | 1859 | 4.18 | **-2.62** | **8.65** |
| Other | 827 | 0.85 | 112 | 0.32 | 987 | 1.02 | 129 | 0.29 | **19.35** | **15.18** |

The unadjusted data looking at the changes of the obese and overweight groups by demographic category.
